# Supplementary material for: Hybrid Models and Biological Model Reduction with PyDSTool
Source: PLoS Comput Biol. 2012 Aug 9;8(8):e1002628. doi: 10.1371/journal.pcbi.1002628 (PMC3415397; doi:10.1371/journal.pcbi.1002628)
Supplement: Text S4 — Complete source code for the PyDSTool package (version 0.88.120504). Includes API documentation and help files linking to web pages. This file is identical to the current public release on Sourceforge.net. (ZIP) [file pcbi.1002628.s004.zip › PyDSTool/html/PyDSTool.Toolbox.FSM.FSM-class.html]

xml version="1.0" encoding="ascii"?


PyDSTool.Toolbox.FSM.FSM


| Home | Trees | Indices | Help | PyDSTool |
| --- | --- | --- | --- | --- |

Package PyDSTool ::
Package Toolbox ::
Module FSM ::
Class FSM

[hide private]

[frames] | no frames]

# Class FSM

source code

```
object --+
         |
        FSM
```

Known Subclasses:
:   - ObjFSM

---

This is a Finite State Machine (FSM).


|  |  |
| --- | --- |
| Instance Methods | [hide private] |

|  |  |
| --- | --- |
| \_\_init\_\_(self, initial\_state, memory=None)  This creates the FSM. | source code |

|  |  |
| --- | --- |
| reset(self)  This sets the current\_state to the initial\_state and sets input\_symbol to None. | source code |

|  |  |
| --- | --- |
| add\_transition(self, input\_symbol, state, action=None, next\_state=None)  This adds a transition that associates: | source code |

|  |  |
| --- | --- |
| add\_transition\_list(self, list\_input\_symbols, state, action=None, next\_state=None)  This adds the same transition for a list of input symbols. | source code |

|  |  |
| --- | --- |
| add\_transition\_any(self, state, action=None, next\_state=None)  This adds a transition that associates: | source code |

|  |  |
| --- | --- |
| set\_default\_transition(self, action, next\_state)  This sets the default transition. | source code |

|  |  |
| --- | --- |
| get\_transition(self, input\_symbol, state)  This returns (action, next state) given an input\_symbol and state. | source code |

|  |  |
| --- | --- |
| process(self, input\_symbol)  This is the main method that you call to process input. | source code |

|  |  |
| --- | --- |
| process\_list(self, input\_symbols)  This takes a list and sends each element to process(). | source code |

**Inherited from `object`**:
`__delattr__`,
`__getattribute__`,
`__hash__`,
`__new__`,
`__reduce__`,
`__reduce_ex__`,
`__repr__`,
`__setattr__`,
`__str__`


|  |  |
| --- | --- |
| Properties | [hide private] |

**Inherited from `object`**:
`__class__`


|  |  |
| --- | --- |
| Method Details | [hide private] |

|  |  |
| --- | --- |
| \_\_init\_\_(self, initial\_state, memory=None)  *(Constructor)* | source code |

This creates the FSM. You set the initial state here. The
"memory" attribute is any object that you want to pass along to
the action functions. It is not used by the FSM. For parsing you would
typically pass a list to be used as a stack.

Overrides: object.\_\_init\_\_

|  |  |
| --- | --- |
| reset(self) | source code |

This sets the current\_state to the initial\_state and sets input\_symbol
to None. The initial state was set by the constructor \_\_init\_\_().

|  |  |
| --- | --- |
| add\_transition(self, input\_symbol, state, action=None, next\_state=None) | source code |

```
This adds a transition that associates:

        (input_symbol, current_state) --> (action, next_state)

The action may be set to None in which case the process() method will
ignore the action and only set the next_state. The next_state may be
set to None in which case the current state will be unchanged.

You can also set transitions for a list of symbols by using
add_transition_list().
```

|  |  |
| --- | --- |
| add\_transition\_list(self, list\_input\_symbols, state, action=None, next\_state=None) | source code |

This adds the same transition for a list of input symbols. You can
pass a list or a string. Note that it is handy to use string.digits,
string.whitespace, string.letters, etc. to add transitions that match
character classes.

The action may be set to None in which case the process() method will
ignore the action and only set the next\_state. The next\_state may be set
to None in which case the current state will be unchanged.

|  |  |
| --- | --- |
| add\_transition\_any(self, state, action=None, next\_state=None) | source code |

```
This adds a transition that associates:

        (current_state) --> (action, next_state)

That is, any input symbol will match the current state.
The process() method checks the "any" state associations after it first
checks for an exact match of (input_symbol, current_state).

The action may be set to None in which case the process() method will
ignore the action and only set the next_state. The next_state may be
set to None in which case the current state will be unchanged.
```

|  |  |
| --- | --- |
| set\_default\_transition(self, action, next\_state) | source code |

This sets the default transition. This defines an action and
next\_state if the FSM cannot find the input symbol and the current state
in the transition list and if the FSM cannot find the current\_state in
the transition\_any list. This is useful as a final fall-through state for
catching errors and undefined states.

The default transition can be removed by setting the attribute
default\_transition to None.

|  |  |
| --- | --- |
| get\_transition(self, input\_symbol, state) | source code |

```
This returns (action, next state) given an input_symbol and state.
This does not modify the FSM state, so calling this method has no side
effects. Normally you do not call this method directly. It is called by
process().

The sequence of steps to check for a defined transition goes from the
most specific to the least specific.

1. Check state_transitions[] that match exactly the tuple,
    (input_symbol, state)

2. Check state_transitions_any[] that match (state)
    In other words, match a specific state and ANY input_symbol.

3. Check if the default_transition is defined.
    This catches any input_symbol and any state.
    This is a handler for errors, undefined states, or defaults.

4. No transition was defined. If we get here then raise an exception.
```

|  |  |
| --- | --- |
| process(self, input\_symbol) | source code |

This is the main method that you call to process input. This may cause
the FSM to change state and call an action. This method calls
get\_transition() to find the action and next\_state associated with the
input\_symbol and current\_state. If the action is None then the action is
not called and only the current state is changed. This method processes
one complete input symbol. You can process a list of symbols (or a
string) by calling process\_list().

|  |  |
| --- | --- |
| process\_list(self, input\_symbols) | source code |

This takes a list and sends each element to process(). The list may be
a string or any iterable object.

  


| Home | Trees | Indices | Help | PyDSTool |
| --- | --- | --- | --- | --- |

|  |  |
| --- | --- |
| Generated by Epydoc 3.0.1 on Fri May 4 15:24:08 2012 | http://epydoc.sourceforge.net |
